# Effect of High-Density Lipoprotein from Healthy Subjects and Chronic Kidney Disease Patients on the CD14 Expression on Polymorphonuclear Leukocytes

**DOI:** 10.3390/ijms22062830

**Published:** 2021-03-11

**Authors:** Gerald Cohen

**Affiliations:** Department of Nephrology and Dialysis, Medical University of Vienna, A-1090 Vienna, Austria; gerald.cohen@meduniwien.ac.at

**Keywords:** high-density lipoprotein, polymorphonuclear leukocytes, CD14, serum amyloid A, lipid rafts, Rac1, immunology, inflammation

## Abstract

In uremic patients, high-density lipoprotein (HDL) loses its anti-inflammatory features and can even become pro-inflammatory due to an altered protein composition. In chronic kidney disease (CKD), impaired functions of polymorphonuclear leukocytes (PMNLs) contribute to inflammation and an increased risk of cardiovascular disease. This study investigated the effect of HDL from CKD and hemodialysis (HD) patients on the CD14 expression on PMNLs. HDL was isolated using a one-step density gradient centrifugation. Isolation of PMNLs was carried out by discontinuous Ficoll-Hypaque density gradient centrifugation. CD14 surface expression was quantified by flow cytometry. The activity of the small GTPase Rac1 was determined by means of an activation pull-down assay. HDL increased the CD14 surface expression on PMNLs. This effect was more pronounced for HDL isolated from uremic patients. The acute phase protein serum amyloid A (SAA) caused higher CD14 expression, while SAA as part of an HDL particle did not. Lipid raft disruption with methyl-β-cyclodextrin led to a reduced CD14 expression in the absence and presence of HDL. HDL from healthy subjects but not from HD patients decreased the activity of Rac1. Considering the known anti-inflammatory effects of HDL, the finding that even HDL from healthy subjects increased the CD14 expression was unexpected. The pathophysiological relevance of this result needs further investigation.

## 1. Introduction

Besides the typical role in reverse cholesterol transport [1], high-density lipoprotein (HDL) has strong anti-inflammatory, anti-oxidative and antithrombotic effects [2], which contribute to immunoregulation [3,4] and a reduced cardiovascular risk [5,6]. However, in inflammatory diseases such as chronic kidney disease (CKD), rheumatoid arthritis, diabetes and coronary artery disease, HDL loses its anti-inflammatory features because of changes in protein and lipid composition [5,7,8]. For example, in studies of vascular smooth muscle cells [9] and on monocytes and dendritic cells [10] a decreased or eliminated anti-inflammatory potential of HDL isolated from hemodialysis (HD) patients was observed. The acute phase protein serum amyloid A (SAA), which induces the expression of inflammatory cytokines in human monocytes, is enriched HDL isolated from CKD patients and patients undergoing HD treatment [10].

Polymorphonuclear leukocytes (PMNLs) are cells of the non-specific immune defense. They are part of the primary immune response and are named after their lobulated nuclei. PMNLs are also commonly referred to as granulocytes because of multiple granules in their cytoplasm. Neutrophils make up the largest group of PMNLs and play an essential role in the defense against bacterial and fungal infections. Dysfunctional PMNLs lead to an increased risk of bacterial infections and cardiovascular disease and are a major source of the increased risk of morbidity and mortality among CKD patients [11]. HDL of CKD and HD patients significantly reduced PMNL apoptosis, an effect that is typically observed with pro-inflammatory substances [12]. In contrast, HDL from healthy subjects had no effect on PMNL apoptotic cell death. HDL from healthy subjects reduced the activation of CD11b surface expression on PMNLs, a first step of the pathogenesis of vascular damage [13,14], whereas HDL from CKD and HD patients did not have this effect. These results suggest that HDL may exacerbate systemic inflammation in uremia.

CD14 is a glycosylphosphatidylinositol-anchored receptor that is located on the cell surface and in the endosomal compartment. It can be expressed as a cell membrane or secreted protein [15]. CD14 is a co-receptor of toll-like receptors (TLRs). TLRs are pattern recognition receptors that detect highly preserved motifs on pathogens (pathogen-associated molecular patterns) and substances that are released by damaged cells (damage-associated molecular patterns) [16]. CD14 can also function as a pattern recognition receptor [17]. While peripheral blood monocytes express over 100,000 CD14 receptors, PMNLs have less than 10,000 on their surface [18]. In PMNLs, CD14 is stored intracellularly as a preformed protein. Upon stimulation and in patients with severe bacterial infections, CD14 surface expression is strongly increased [19]. In this study, the effects of HDL from healthy subjects as well as from CKD and HD patients on the CD14 surface expression on PMNLs were analyzed.

## 2. Results

### 2.1. Effect of HDL on CD14 Surface Expression

**Figure 1:**
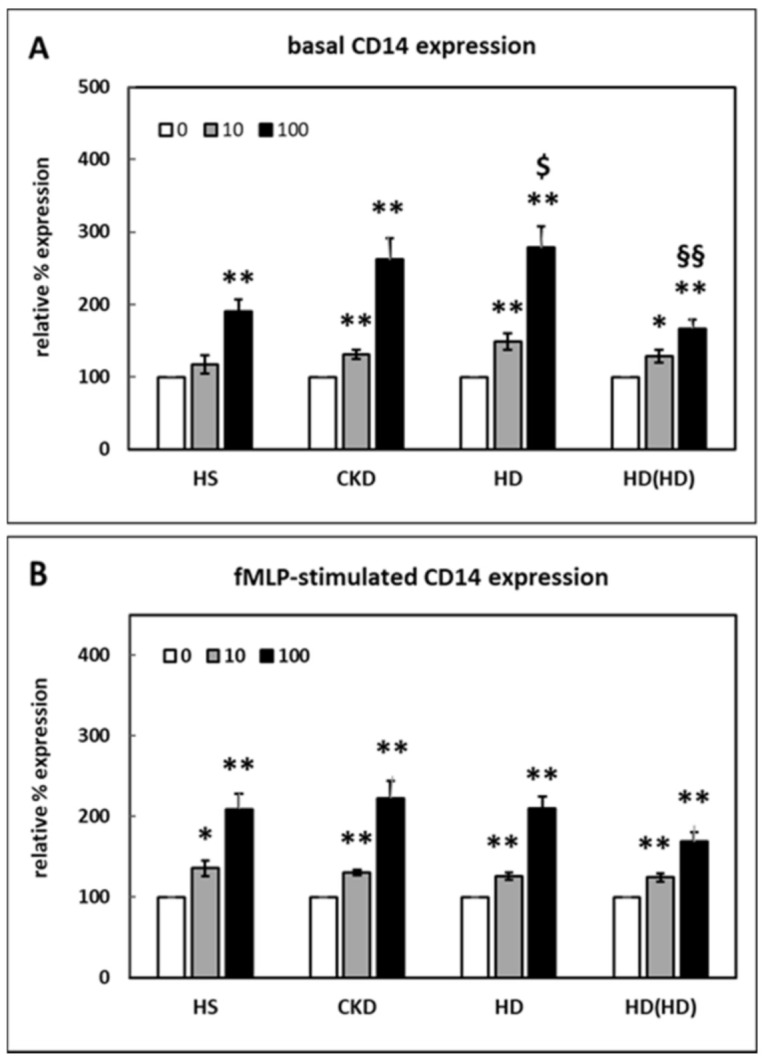
Effect of high-density lipoprotein (HDL) from healthy subjects (HS), *n* = 7, chronic kidney disease (CKD) patients stage 3 and 4 (CKD), *n* = 18, hemodialysis (HD) patients (HD), *n* = 8, on polymorphonuclear leukocytes (PMNLs) from healthy subjects at 0, 10 and 100 µg/mL final concentration. HD(HD): Effect of HDL from HD patients on PMNLs from HD patients, *n* = 9; the percentage of CD14 positive cells in the absence of HDL was set as 100%. There was no statistically significant difference between the absolute values. (**A**) Basal CD14 surface expression. (**B**) CD14 surface expression stimulated by N-formyl-methionyl-leucyl-phenylalanine (fMLP). * *p* < 0.05 and ** *p* < 0.01 versus 0 µg/mL HDL; $ *p* < 0.05 versus HS; §§ *p* < 0.01 versus HD; data shown are mean values ± standard error of the mean (SEM).

HDL increased the basal CD14 expression (Figure 1A). This change was significant except for HDL isolated from healthy subjects (HS-HDL) at a final concentration of 10 µg/mL.

At 100 µg/mL, the CD14 expression was higher for HDL from CKD- and HD patients compared to HS-HDL. This difference reached statistical significance only for HDL from HD patients (HD-HDL). Considering that HDL is known to be an anti-inflammatory factor, this activating effect was unexpected. Compared to HS-HDL, however, uremic HDL induced a stronger stimulation of CD14 expression.

The columns HS, CKD and HD in Figure 1 represent the effect of the corresponding HDL isolates on PMNLs isolated from healthy individuals. However, blood cells from patients with end-stage renal disease are continuously exposed to the uremic milieu and may not show the same responsiveness. Therefore, the effect of HD-HDL on PMNLs from patients undergoing HD treatment was tested. As indicated by the columns HD(HD), HD-HDL increased the CD14 expression on cells from HD patients to a lesser extent than on PMNLs from healthy probands (Figure 1A).

As previously shown, N-formyl-methionyl-leucyl-phenylalanine (fMLP), like lipopolysaccharide (LPS), significantly increases CD14 cell surface expression on PMNLs [20]. The fMLP-stimulated CD14 expression was significantly increased in the presence of all HDL-isolates at both concentrations tested (Figure 1B). There were no statistically significant differences between these groups.

The absolute percentage of the basal CD14 expression on PMNLs from healthy subjects (HS) was 1.80 ± 0.21% and on PMNLs from HD patients 2.34 ± 0.47%. The absolute percentage of the fMLP-stimulated CD14 expression on PMNLs from HS was 5.67 ± 0.61% and on PMNLs from HD patients 4.81 ± 1.07%. Both differences were not statistically significant.

### 2.2. Effect of SAA on CD14 Surface Expression

**Figure 2:**
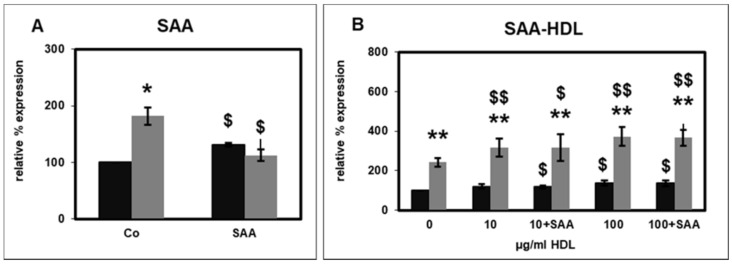
Effect of serum amyloid A protein (SAA) at a final concentration of 10 µg/mL ((**A**); *n* = 3) and of HDL isolated from healthy subjects (HS-HDL) and HS-HDL spiked with SAA (+) at final concentrations of 10 µg/mL and 100 µg/mL ((**B**); *n* = 8) on the basal (black bars) and fMLP-stimulated (grey bars) CD14 surface expression. The percentage of CD14 positive cells in the absence of SAA and fMLP (Co: buffer as control, 0 µg/mL HDL) was set as 100%. * *p* < 0.05 and ** *p* < 0.01 versus unstimulated values; $ *p* < 0.05, $$ *p* < 0.01 versus control (Co: 0.01% bovine serum albumin) for A, versus 0 µg/mL HDL for B; data shown are mean values ± standard error of the mean (SEM).

SAA, which accumulates in HDL particles from uremic patients, induces pro-inflammatory cytokine production in human monocytes [10]. In PMNLs, pre-incubation with SAA significantly increased the basal CD14 expression, while stimulation by fMLP was completely abolished (Figure 2A), indicating a desensitization of the fMLP receptor by SAA.

On the other hand, the influence of HS-HDL, which was spiked with SAA, on PMNL CD14 expression did not differ from that of HS-HDL (Figure 2B). Hence, incorporation of SAA into HDL shielded the effect of SAA.

### 2.3. Effect of Lipid Raft Disruption on CD14 Surface Expression

**Figure 3:**
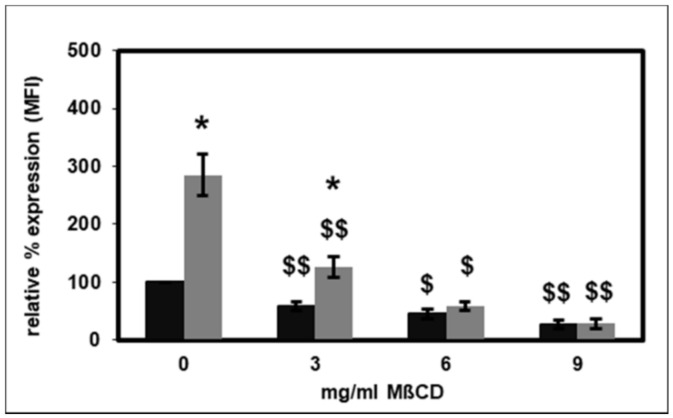
Effect of methyl-β-cyclodextrin (MβCD) on basal (black bars) and fMLP-stimulated (grey bars) CD14 surface expression. The unstimulated CD11b expression measured as mean fluorescence intensity (MFI) in the absence of MβCD was set as 100%. *n* = 5 for 0 and 3 mg/mL MβCD; *n* = 3 for 6 and 9 mg/mL MβCD. * *p* < 0.05 versus the unstimulated values; $ *p* < 0.05 and $$ *p* < 0.01 versus the absence of MβCD; data shown are mean values ± SEM.

HDL can alter cell functions by reducing the membrane cholesterol content, particularly within lipid rafts [21]. In this study, the effect of selective destruction of lipid rafts on PMNL CD14 expression by using methyl-β-cyclodextrin (MβCD) to disintegrate lipid rafts was evaluated [22]. MβCD significantly reduced both basal and fMLP-stimulated CD14 expression in a concentration-dependent manner (Figure 3). At 6 and 9 mg/mL MβCD, the stimulation by fMLP was completely abolished.

**Figure 4:**
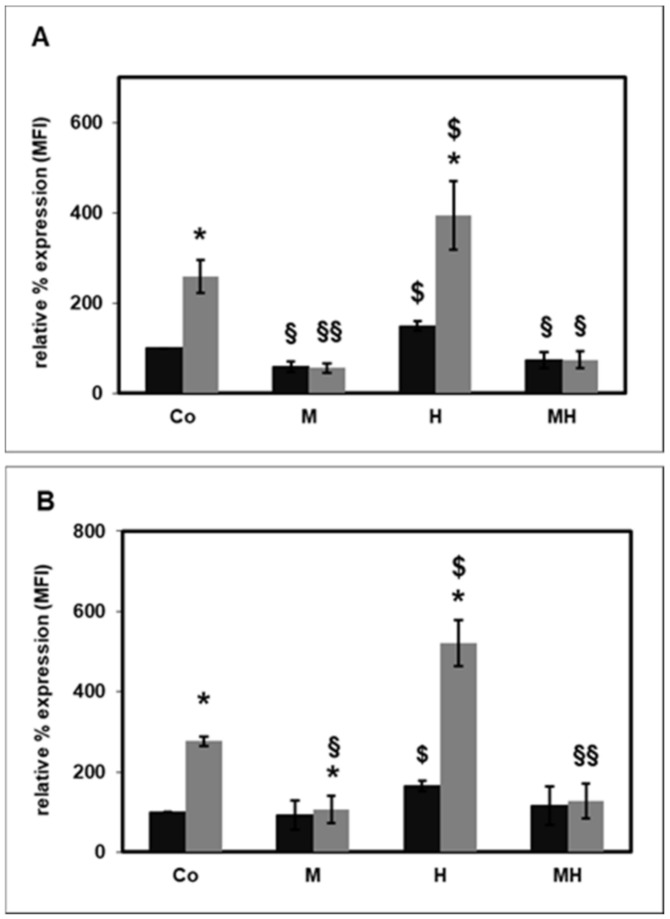
Effect of MβCD (M; 6 mg/mL) on basal (black bars) and fMLP-stimulated (grey bars) CD14 surface expression in the absence and presence of 100 µg/mL HDL (H) from healthy subjects ((**A**); *n* = 4) and from HD patients ((**B**); *n* = 5). The unstimulated CD11b expression measured as mean fluorescence intensity (MFI) in the absence of MβCD was set as 100%. * *p* < 0.05 versus the unstimulated values; § *p* < 0.05 and §§ *p* < 0.01 versus the absence of MβCD; $ *p* < 0.05 versus the absence of HDL; data shown are mean values ± SEM.

Next, the effect of MβCD on the CD14 expression in the presence of HS-HDL (Figure 4A) or HD-HDL (Figure 4B) was tested. Neither HS-HDL nor HD-HDL changed the effect of MβCD on the basal or fMLP-stimulated CD14 expression. On the other hand, MβCD completely eliminated the HDL-induced CD14 expression.

### 2.4. Effect of HDL on Rac1 Activity

**Figure 5:**
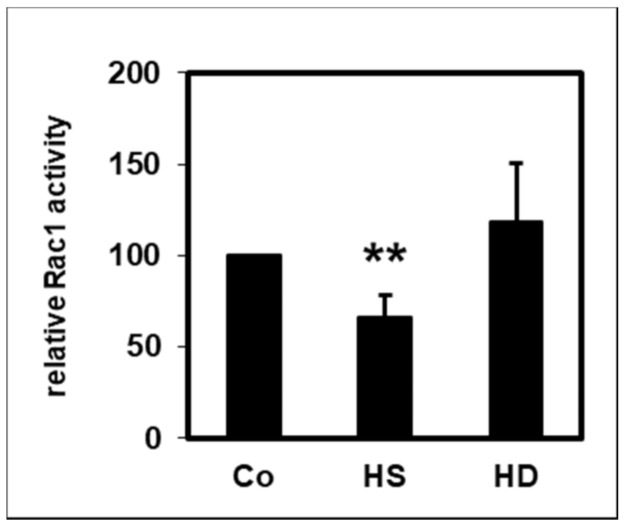
Effect of HDL (10 µg/mL) from healthy subjects (HS), *n* = 9 and HD patients (HD), *n* = 7, on the basal Rac1 activity of PMNLs from healthy subjects. The activity in the absence of HDL was set as 100%. ** *p* < 0.01 versus control; data shown are mean values ± SEM.

The small 21 kDa GTPase Rac1 controls many different cellular processes and contributes to the inflammation associated with kidney disease [23]. Like CD14, it is partly located in lipid rafts [24]. Measurement of the active GTP-bound form of Rac1 showed that HS-HDL significantly reduced the basal Rac1 activity, while HD-HDL did not show this attenuating effect (Figure 5).

## 3. Discussion

Polymorphonuclear leukocytes (PMNLs) have binding sites for HDL [25], an anti-inflammatory factor and for its main apolipoprotein, ApoA-I [26]. In this study, the effect of HDL from healthy subjects and from CKD and HD patients on the CD14 expression of PMNLs was investigated. HDL increased the surface expression of CD14 on PMNLs from healthy subjects. This effect was more pronounced for HDL isolated from uremic patients. Whereas the acute phase protein SAA led to an increased CD14 expression, SAA did not show this effect when it was part of an HDL particle. The disruption of lipid rafts resulted in a reduced CD14 expression in the absence as well as in the presence of HDL. HDL from healthy subjects, but not from HD patients, decreased the activity of the small GTPase Rac1.

Chronic kidney disease (CKD) is one of the leading health problems worldwide and causes high economic costs to the health system [27]. Inflammation-related cardiovascular disease and infections are the two major reasons for the high risk of morbidity and mortality among CKD patients. Since it was discovered that HDL developed inflammatory properties in CKD patients, one research focus became the effect of uremic HDL on immune cells. We previously described the effects of HDL, isolated from patients with CKD and patients undergoing HD treatment, on various functions of PMNLs, which are crucial elements of the non-specific cellular immune defense. We concluded that HDL contributes to systemic inflammation in uremic patients by disturbing PMNL functions. The effect of HDL on CD14 expression has not yet been reported.

The expression of CD14, a glycosylphosphatidylinositol-anchored co-receptor of TLRs, is much lower on the surface of PMNLs compared to monocytes [18]. However, PMNLs express high levels of CD14 mRNA [28]. In PMNLs, CD14 is stored intracellularly in azurophilic granules and plasma membrane-secretory vesicles [20]. The activation of PMNLs during bacterial infections in vivo [19] and by stimulants such as LPS and fMLP in vitro [20] leads to a strong increase in CD14 cell surface expression.

In PMNLs of patients with myelodysplastic syndromes, stem cell malignancies with an increased risk of developing acute myeloid leukemia deficient in formin proteins involved in linear actin polymerization show a specific increase of CD14 messenger RNA [29]. In contrast to PMNLs, this upregulation is not observed in other cell lineages, which shows that PMNLs differ from monocytes with regard to CD14 expression. The aberrant overexpression of CD14 on PMNLs sensitizes the innate immune response [29]. As shown in Figure 1, HDL significantly increased the expression of CD14 on PMNLs. This finding was unexpected given the known anti-oxidative and anti-inflammatory effects of HDL [2]. Nevertheless, HDL from CKD and HD patients caused a more pronounced increase in CD14 expression compared to HDL from healthy subjects (Figure 1). Further studies are needed to elucidate the significance of this finding.

The highest HDL concentration used in the assays was 100 µg protein per mL, a concentration below the physiological level. In view of a possible ceiling effect, higher HDL concentrations do not necessarily have a more pronounced impact. However, the main, even though unexpected, finding that HDL increases the CD14 expression on PMNLs would not change if a plateau effect occurs. The highest final HDL concentration used in this study (100 µg/mL) has also been used in earlier in vitro investigations to analyze the HDL effect [10,12]. In studies testing the in vitro effect of HDL, the HDL concentration is usually given as µg HDL protein per mL, whereas clinical medicine uses mg HDL cholesterol per dL. The physiological HDL levels range from 30 to 80 mg HDL cholesterol/dL. Based on results of studies examining the HDL composition, 1000 mg HDL protein corresponds to 370–380 mg HDL cholesterol [30,31]. Therefore, a concentration of 100 µg HDL protein per mL corresponds to approximately 3.75 mg HDL cholesterol per dL.

Changes in the protein composition of HDL have a substantial influence on the cardioprotective properties of HDL [32]. The uremic milieu, which is characterized by the accumulation of uremic toxins, causes qualitative changes of HDL in patients with impaired kidney function. The enrichment of the uremic toxins such as symmetric dimethylarginine in HDL contributes to the adverse effect of HDL in uremic patients [33]. Using shotgun proteomics, Weichhart et al. [10] showed that SAA is enriched in HDL from HD patients. HDL-associated SAA was already present in healthy controls but was markedly elevated in CKD4 patients as shown by immunoblotting. Wang et al. [34] investigated the effect of the HD procedure on the HDL proteome and found that, compared to patients with advanced CKD, recent HD initiation is associated with an even greater relative abundance of HDL-associated proteins related to lipid metabolism and inflammation such as SAA. They concluded that CKD and HD might uniquely affect the HDL proteome, thereby generating different versions of dysfunctional HDL.

As a result of SAA accumulation on the HDL particle, HD-HDL loses its anti-inflammatory properties and even becomes pro-inflammatory [9,10,35]. SAA is an important acute phase protein that is produced by the liver under inflammatory conditions. It has 104 amino acids and occurs in low levels (20–50 µg/mL) in the serum of healthy individuals, but increases by a factor of 1000 24 h after the onset of an acute phase response [36]. SAA levels and cardiovascular mortality are significantly associated in patients with high cardiovascular risk [37]. The SAA incorporated in the HDL particle is linked to cardiac events independently of HDL-cholesterol serum levels [38].

In human monocytes, SAA incorporated into HDL abolished the anti-inflammatory effect of HDL [10]. SAA and SAA-conjugated HDL stimulate the formation of macrophage foam cells [39]. In PMNLs, SAA significantly increased the basal CD14 expression (Figure 2A). Furthermore, SAA completely abrogated the stimulation of CD14 expression by fMLP (Figure 2A). This effect could be a result of desensitization of the fMLP receptor by SAA. SAA activates several receptors, for instance, the formyl peptide receptor 2 (FPR2), the toll-like receptors TLR2 and TLR4, the scavenger receptor SR-BI and the ATP receptor P2X7 [40]. Receptor desensitization is a mechanism for discrimination between the multiple signals PMNLs receive during inflammation. The desensitization can be homologous, as in the case of fMLP-induced mobilization intracellular calcium stores, or heterologous, e.g., between the fMLP and C5a receptor [41].

The SAA effect on PMNL CD14 expression was not observed when SAA was incorporated into HDL, indicating that the SAA effect was masked by HDL (Figure 2B). Consistent with this observation, Shridas et al. [42] demonstrated that the integration of SAA in HDL abolished the inflammasome activation and ROS generation mediated by SAA. On the other hand, both SAA and HDL-conjugated SAA can stimulate the formation of macrophage foam cells [39]. Furthermore, lipid-poor, but not HDL-linked SAA can induce the production of pro-inflammatory cytokines in a monocyte cell line [43]. While recombinant SAA induces chemotaxis of PMNLs and monocytes, this effect is blocked by pre-incubating SAA with HDL [44].

Lipid rafts are cholesterol- and sphingolipid-rich plasma membrane regions that provide specific lipid environments that regulate the organization and function of several plasma membrane proteins [24] and serve as a platform for receptors on the cell surface [45]. Lipid–lipid, lipid–protein and protein–protein interactions contribute to lipid raft formation and their stabilization. Lipid rafts have a diameter from <70 nm to 2 µm and cover 13 to 50% of the cell surface [46]. Their number, structure and distribution control basic biological activities, including immune reactions [47], signaling cascades [48] and cell migration and adhesion [49]. Lipid rafts provide a suitable microenvironment for CD14-dependent receptor clustering, which is involved in innate immunity and induces specific co-assembly of further receptors [50].

The effect of selective destruction of lipid rafts on the CD14 surface expression on PMNLs was investigated using MβCD [22]. As expected, MβCD significantly reduced the basal and fMLP-stimulated CD14 expression (Figure 3). HDL has been reported to change cell functions by lowering the membrane cholesterol content, particularly in lipid rafts [21]. In patients with diabetes mellitus, the CD14 expression on monocytes is inversely correlated with HDL cholesterol levels [51]. ApoA-I stimulated cholesterol efflux from monocyte-derived macrophages, which led to cholesterol depletion, disruption of lipid rafts and decreased the expression of CD14 [52]. In contrast to these findings, data from the present study indicate that HDL caused an augmented CD14 surface expression on PMNLs (Figure 1). Therefore, another mechanism yet to be identified must be responsible for this increase. Interestingly, Olsson et al. reported that CD14 was not strictly associated with lipid rafts. CD14 was only present in lipid rafts after LPS stimulation, but not in unstimulated cells of a macrophage cell line [53]. Moreover, as shown in Figure 4, HDL not only did not change this effect of MβCD on the basal or fMLP-stimulated CD14 expression, MβCD even completely abolished the increased CD14 expression caused by HDL. Therefore, lipid rafts seem to be necessary for the elevated HDL-induced CD14 expression.

The small 21 kDa GTPase Rac1 belongs to the Rho family of GTPases and regulates many diverse cellular processes, including the cell cycle, cell–cell adhesion and motility. Rac1 is involved in several cardiovascular pathologies, e.g., vascular smooth muscle proliferation, atherosclerosis cardiomyocyte hypertrophy and endothelial dysfunction in hypertension, and is considered a promising therapeutic target in cerebro- and cardiovascular diseases [54]. In the host defense functions of PMNLs, Rac-GTPases play a central role in regulating the expression of adhesion molecules and the orchestration of neutrophil functions, which are necessary for the destruction of invading microorganisms [55]. Like CD14, Rac1 is also partially associated with lipid rafts [24]. Activation of Rac1 involves translocation to membranes at domain boundaries, followed by diffusion of Rac1 into raft and non-raft domains [56]. PMNLs express more Rac1 than monocytes, which indicates a high need for Rho GTPase activity, which is necessary for a fast immune response [57]. In vascular smooth muscle cells, HDL reduces the activation of Rac1, which is necessary for the activation of the NAD(P)H-oxidase according to its anti-oxidative effect [58]. In this study, measurement of the active GTP-bound form of Rac1 showed that HDL significantly reduced the basal Rac1 activity in PMNLs (Figure 5). However, this attenuating influence was observed only for HS-HDL but not for HD-HDL.

The data presented here show that HDL from CKD and HD patients increases the CD14 expression on PMNLs. Notably, the incubation of PMNLs with HDL from healthy subjects has the same effect, albeit less pronounced. This finding was quite surprising given the known anti-oxidative and anti-inflammatory effects of HDL [2]. The pathophysiological implication of this result requires further research.

## 4. Materials and Methods

### 4.1. Patients

The ethics committee of the General Hospital Vienna approved this study according to the declaration of Helsinki (EK 980/2011). Informed consent was obtained from all participants. Subjects with infection and intercurrent illness were excluded from this study.

In a recent publication, the effect of HDL on PMNL apoptosis, CD11b surface expression, oxidative burst and chemotaxis was assessed [12]. HDL samples from the same pool of patients and probands were used in this study (see Supplementary Tables S1 and S2; Both tables were taken from reference [12]). The pooled data of HDL from stage 3 and stage 4 CKD patients are presented because they did not differ in the effects assessed in this study. HD patients were dialyzed on standard bicarbonate basis for 4 to 5 h three times a week with biocompatible polysulfone HD membranes (Fresenius, Bad Homburg, Germany). The Kt/V values of the patients were 1.2. All HD patients were without residual renal function.

### 4.2. High-Density Lipoprotein Isolation

A one-step density gradient centrifugation was used to isolate HDL as previously described [59]. Blood was collected in ethylene diamine tetra-acetic acid (EDTA) tubes. The density of the plasma was adjusted to 1.24 g/mL with potassium bromide (Sigma-Aldrich, St. Louis, MO, USA). Four mL of plasma was layered under phosphate-buffered saline (PBS, pH 7.4; BioWhittaker Lonza Services, Verviers, Belgium) with a density of 1.06 g/mL in a polyallomer centrifuge tube. After centrifugation in a fixed-angle type 75 Ti rotor in an Optima L-80 ultracentrifuge (Beckman Coulter, Fullerton, CA, USA) at 60,000 rpm (371,000 g) at 15° C for 5 h, the fractions containing HDL were collected, desalted to PBS (polyacrylamide 6000 desalting column; Thermo Scientific, Rockford, IL, USA) and stored at −80 °C. HDL from each group (control, CKD stage 3 and 4 and HD patients) has been tested individually. The concentration of the isolated HDL was determined by the bicinchoninic acid protein assay (Pierce, Rockford, IL, USA). The concentration of HDL is shown as µg protein per mL.

HDL spiked with serum amyloid A (SAA-HDL) was prepared as previously described [10,60]. Eight milliliters of plasma from a healthy individual together with 50 µg SAA (Preprotech, Rocky Hill, NL, USA) in PBS or PBS alone as control were incubated for 3 h at 4 °C. For the isolation of SAA-HDL, the same procedure as for HDL was used.

### 4.3. Isolation of Polymorphonuclear Leukocytes

PMNLs were isolated from heparinized blood by discontinuous Ficoll-Hypaque (GE Healthcare Bio-Sciences AB, Uppsala, Sweden) density gradient centrifugation and hypotonic lysis of erythrocytes as previously described [61]. As determined by ethidium bromide exclusion (GibcoBRL Life Technologies, Gaithersburg, MD, USA), the viability of PMNLs using this protocol was over 95%.

### 4.4. Surface CD14 Expression

Ten microliters of HDL of a tenfold concentrated stock solution was added to 90 µL PMNL suspension (0.3 × 10^6^ cells/mL). After incubation for 30 min at 37°, 10 µL PBS or fMLP (Sigma-Aldrich Chemie GmbH, Steinheim, Germany) stock solution (10^−7^ M) was added and incubated for a further 30 min at 37 °C. The samples were incubated for 45 min at room temperature in the presence of a fluorescence-labelled monoclonal antibody (ECD-anti-CD14; Immunotech Beckman Coulter, Marseille, France) and then placed on ice. After addition of 500 µL ice cold PBS, flow cytometry was performed on an Epics XL-MCL (Coulter, Hialeah, FL, USA). The surface expression was measured as percentage of CD14 positive cells.

### 4.5. Lipid Raft Disintegration

To test the influence of selective lipid raft disintegration on PMNL functions, MβCD (Sigma Life Science, Sigma-Aldrich Chemie GmbH) was used as previously described [18].

### 4.6. Rac1 Activity

The Rac1 activity was determined using the Rac1 activation pull-down assay from Cytoskeleton Inc. (Denver, CO, USA). This test is based on the specific binding of the GTP-bound, i.e., active form of Rac1 to affinity beads and the determination of the amount of activated Rac1 by Western blotting using a Rac1 specific antibody.

PMNLs isolated from HS were incubated at 37 °C in the presence of HDL from HS or HD patients for 15 min. The preparation of the cell lysates and the isolation of activated Rac1 were performed according to the instructions of the supplier. The samples were shock-frozen in liquid nitrogen and stored at −80 °C until application to gel electrophoresis and Western blotting. Western blotting and the quantification of the intensity of the bands were performed as previously described [62].

### 4.7. Statistical Analysis

The Wilcoxon matched-pair signed-rank test was used to analyze data from at least six independent experiments. When fewer than six independent experiments were performed, data were analyzed by the paired two-tailed t-test. Data presented are mean values ± standard error of the mean (SEM).

## Data Availability

The data presented in this study are available on request from the author.

## Supplementary Materials

The following are available online at https://www.mdpi.com/1422-0067/22/6/2830/s1.

## References

[1] Rosenson R.S., Brewer H.B., Davidson W.S., Fayad Z.A., Fuster V., Goldstein J., Hellerstein M., Jiang X.C., Phillips M.C., Rader D.J. (2012). Cholesterol efflux and atheroprotection: Advancing the concept of reverse cholesterol transport. Circulation.

[2] Navab M., Reddy S.T., Van Lenten B.J., Fogelman A.M. (2011). HDL and cardiovascular disease: Atherogenic and atheroprotective mechanisms. Nat. Rev. Cardiol..

[3] Creasy K.T., Kane J.P., Malloy M.J. (2018). Emerging roles of HDL in immune function. Curr. Opin. Lipidol..

[4] Norata G.D., Pirillo A., Ammirati E., Catapano A.L. (2012). Emerging role of high density lipoproteins as a player in the immune system. Atherosclerosis.

[5] Pirillo A., Catapano A.L., Norata G.D. (2018). Biological Consequences of Dysfunctional HDL. Curr. Med. Chem..

[6] Rye K.A., Barter P.J. (2014). Cardioprotective functions of HDLs. J. Lipid Res..

[7] Marsche G., Saemann M.D., Heinemann A., Holzer M. (2013). Inflammation alters HDL composition and function: Implications for HDL-raising therapies. Pharmacol. Ther..

[8] Saemann M.D., Poglitsch M., Kopecky C., Haidinger M., Horl W.H., Weichhart T. (2010). The versatility of HDL: A crucial anti-inflammatory regulator. Eur. J. Clin. Investig..

[9] Tolle M., Huang T., Schuchardt M., Jankowski V., Prufer N., Jankowski J., Tietge U.J., Zidek W., Van Der Giet M. (2012). High-density lipoprotein loses its anti-inflammatory capacity by accumulation of pro-inflammatory-serum amyloid A. Cardiovasc. Res..

[10] Weichhart T., Kopecky C., Kubicek M., Haidinger M., Doller D., Katholnig K., Suarna C., Eller P., Tolle M., Gerner C. (2012). Serum Amyloid A in Uremic HDL Promotes Inflammation. J. Am. Soc. Nephrol..

[11] Haag-Weber M., Hörl W.H. (1996). Dysfunction of polymorphonuclear leukocytes in uremia. Semin. Nephrol..

[12] Raupachova J., Kopecky C., Cohen G. (2019). High-Density Lipoprotein from Chronic Kidney Disease Patients Modulates Polymorphonuclear Leukocytes. Toxins.

[13] Kaysen G.A. (2002). Role of inflammation and its treatment in ESRD patients. Blood Purif..

[14] Crockett-Torabi E., Ward P.A. (1996). The role of leukocytes in tissue injury. Eur. J. Anaesthesiol..

[15] Zanoni I., Granucci F. (2013). Role of CD14 in host protection against infections and in metabolism regulation. Front. Cell Infect. Microbiol..

[16] Leemans J.C., Kors L., Anders H.J., Florquin S. (2014). Pattern recognition receptors and the inflammasome in kidney disease. Nat. Rev. Nephrol..

[17] Zanoni I., Ostuni R., Capuano G., Collini M., Caccia M., Ronchi A.E., Rocchetti M., Mingozzi F., Foti M., Chirico G. (2009). CD14 regulates the dendritic cell life cycle after LPS exposure through NFAT activation. Nature.

[18] Antal-Szalmas P., Strijp J.A., Weersink A.J., Verhoef J., Van Kessel K.P. (1997). Quantitation of surface CD14 on human monocytes and neutrophils. J. Leukoc. Biol..

[19] Wagner C., Deppisch R., Denefleh B., Hug F., Andrassy K., Hansch G.M. (2003). Expression patterns of the lipopolysaccharide receptor CD14, and the FCgamma receptors CD16 and CD64 on polymorphonuclear neutrophils: Data from patients with severe bacterial infections and lipopolysaccharide-exposed cells. Shock.

[20] Rodeberg D.A., Morris R.E., Babcock G.F. (1997). Azurophilic granules of human neutrophils contain CD14. Infect. Immun..

[21] Wang S.H., Yuan S.G., Peng D.Q., Zhao S.P. (2012). HDL and ApoA-I inhibit antigen presentation-mediated T cell activation by disrupting lipid rafts in antigen presenting cells. Atherosclerosis.

[22] Sitrin R.G., Sassanella T.M., Landers J.J., Petty H.R. (2010). Migrating Human Neutrophils Exhibit Dynamic Spatiotemporal Variation in Membrane Lipid Organization. Am. J. Respir. Cell Mol. Biol..

[23] Sedeek M., Nasrallah R., Touyz R.M., Hebert R.L. (2013). NADPH Oxidases, Reactive Oxygen Species, and the Kidney: Friend and Foe. J. Am. Soc. Nephrol..

[24] Gaus K., Rodriguez M., Ruberu K.R., Gelissen I., Sloane T.M., Kritharides L., Jessup W. (2005). Domain-specific lipid distribution in macrophage plasma membranes. J. Lipid Res..

[25] Schmitz G., Wulf G., Bruning T., Assmann G. (1987). Flow-cytometric determination of high-density-lipoprotein binding sites on human leukocytes. Clin. Chem..

[26] Blackburn W.D., Dohlman J.G., Venkatachalapathi Y.V., Pillion D.J., Koopman W.J., Segrest J.P., Anantharamaiah G.M. (1991). Apolipoprotein A-I decreases neutrophil degranulation and superoxide production. J. Lipid Res..

[27] Hill N.R., Fatoba S.T., Oke J.L., Hirst J.A., O’Callaghan C.A., Lasserson D.S., Hobbs F.D. (2016). Global Prevalence of Chronic Kidney Disease—A Systematic Review and Meta-Analysis. PLoS ONE.

[28] Goyert S.M., Ferrero E., Rettig W.J., Yenamandra A.K., Obata F., Le Beau M.M. (1988). The CD14 monocyte differentiation antigen maps to a region encoding growth factors and receptors. Science.

[29] Keerthivasan G., Mei Y., Zhao B., Zhang L., Harris C.E., Gao J., Basiorka A.A., Schipma M.J., McElherne J., Yang J. (2014). Aberrant overexpression of CD14 on granulocytes sensitizes the innate immune response in mDia1 heterozygous del(5q) MDS. Blood.

[30] Nakanishi S., Vikstedt R., Soderlund S., Lee-Rueckert M., Hiukka A., Ehnholm C., Muilu M., Metso J., Naukkarinen J., Palotie L. (2009). Serum, but not monocyte macrophage foam cells derived from low HDL-C subjects, displays reduced cholesterol efflux capacity. J. Lipid Res..

[31] Persegol L., Verges B., Foissac M., Gambert P., Duvillard L. (2006). Inability of HDL from type 2 diabetic patients to counteract the inhibitory effect of oxidised LDL on endothelium-dependent vasorelaxation. Diabetologia.

[32] Shao B., De Boer I., Tang C., Mayer P.S., Zelnick L., Afkarian M., Heinecke J.W., Himmelfarb J. (2015). A Cluster of Proteins Implicated in Kidney Disease Is Increased in High-Density Lipoprotein Isolated from Hemodialysis Subjects. J. Proteome Res..

[33] Zewinger S., Kleber M.E., Rohrer L., Lehmann M., Triem S., Jennings R.T., Petrakis I., Dressel A., Lepper P.M., Scharnagl H. (2017). Symmetric dimethylarginine, high-density lipoproteins and cardiovascular disease. Eur. Heart J..

[34] Wang K., Zelnick L.R., Hoofnagle A.N., Vaisar T., Henderson C.M., Imrey P.B., Robinson-Cohen C., De Boer I.H., Shiu Y.-T., Himmelfarb J. (2018). Alteration of HDL Protein Composition with Hemodialysis Initiation. Clin. J. Am. Soc. Nephrol..

[35] Han C.Y., Tang C., Guevara M.E., Wei H., Wietecha T., Shao B., Subramanian S., Omer M., Wang S., O’Brien K.D. (2016). Serum amyloid A impairs the antiinflammatory properties of HDL. J. Clin. Investig..

[36] Sack G.H. (2018). Serum amyloid A—A review. Mol. Med..

[37] Zewinger S., Drechsler C., Kleber M.E., Dressel A., Riffel J., Triem S., Lehmann M., Kopecky C., Saemann M.D., Lepper P.M. (2015). Serum amyloid A: High-density lipoproteins interaction and cardiovascular risk. Eur. Heart J..

[38] Kopecky C., Genser B., Drechsler C., Krane V., Kaltenecker C.C., Hengstschlager M., Marz W., Wanner C., Saemann M.D., Weichhart T. (2015). Quantification of HDL Proteins, Cardiac Events, and Mortality in Patients with Type 2 Diabetes on Hemodialysis. Clin. J. Am. Soc. Nephrol..

[39] Lee H.Y., Kim S.D., Baek S.-H., Choi J.H., Cho K.H., Zabel B.A., Bae Y.S. (2013). Serum amyloid A stimulates macrophage foam cell formation via lectin-like oxidized low-density lipoprotein receptor 1 upregulation. Biochem. Biophys. Res. Commun..

[40] Ye R.D., Sun L. (2015). Emerging functions of serum amyloid A in inflammation. J. Leukoc. Biol..

[41] Blackwood R.A., Hartiala K.T., Kwoh E.E., Transue A.T., Brower R.C. (1996). Unidirectional heterologous receptor desensitization between both the fMLP and C5a receptor and the IL-8 receptor. J. Leukoc. Biol..

[42] Shridas P., De Beer M.C., Webb N.R. (2018). High-density lipoprotein inhibits serum amyloid A-mediated reactive oxygen species generation and NLRP3 inflammasome activation. J. Biol. Chem..

[43] Kim M.H., De Beer M.C., Wroblewski J.M., Webb N.R., De Beer F.C. (2013). SAA does not induce cytokine production in physiological conditions. Cytokine.

[44] Badolato R., Wang J.M., Murphy W.J., Lloyd A.R., Michiel D.F., Bausserman L.L., Kelvin D.J., Oppenheim J.J., Fava R.A., Olsen N.J. (1994). Serum amyloid A is a chemoattractant: Induction of migration, adhesion, and tissue infiltration of monocytes and polymorphonuclear leukocytes. J. Exp. Med..

[45] Oh H., Mohler E.R., Tian A., Baumgart T., Diamond S.L. (2009). Membrane cholesterol is a biomechanical regulator of neutrophil adhesion. Arterioscler. Thromb. Vasc. Biol..

[46] Pike L.J. (2003). Lipid rafts: Bringing order to chaos. J. Lipid Res..

[47] Harder T., Engelhardt K.R. (2004). Membrane Domains in Lymphocytes—From Lipid Rafts to Protein Scaffolds. Traffic.

[48] Simons K., Toomre D. (2000). Lipid rafts and signal transduction. Nat. Rev. Mol. Cell Biol..

[49] Gomez-Mouton C., Abad J.L., Mira E., LaCalle R.A., Gallardo E., Jimenez-Baranda S., Illa I., Bernad A., Manes S., Martinez A.C. (2001). Segregation of leading-edge and uropod components into specific lipid rafts during T cell polarization. Proc. Natl. Acad. Sci. USA.

[50] Schmitz G., Orso E. (2002). CD14 signalling in lipid rafts: New ligands and co-receptors. Curr. Opin. Lipidol..

[51] Patino R., Ibarra J., Rodriguez A., Yague M.R., Pintor E., Fernandez-Cruz A., Figueredo A. (2000). Circulating monocytes in patients with diabetes mellitus, arterial disease, and increased CD14 expression. Am. J. Cardiol..

[52] Smythies L.E., White C.R., Maheshwari A., Palgunachari M.N., Anantharamaiah G.M., Chaddha M., Kurundkar A.R., Datta G. (2010). Apolipoprotein A-I mimetic 4F alters the function of human monocyte-derived macrophages. Am. J. Physiol. Cell Physiol..

[53] Olsson S., Sundler R. (2006). The role of lipid rafts in LPS-induced signaling in a macrophage cell line. Mol. Immunol..

[54] Carrizzo A., Forte M., Lembo M., Formisano L., Puca A.A., Vecchione C. (2014). Rac-1 as a New Therapeutic Target in Cerebro- and Cardio-Vascular Diseases. Curr. Drug Targets.

[55] Pantarelli C., Welch H.C.E. (2018). Rac-GTPases and Rac-GEFs in neutrophil adhesion, migration and recruitment. Eur. J. Clin. Investig..

[56] Moissoglu K., Kiessling V., Wan C., Hoffman B.D., Norambuena A., Tamm L.K., Schwartz M.A. (2014). Regulation of Rac1 translocation and activation by membrane domains and their boundaries. J. Cell Sci..

[57] Van Helden S.F., Anthony E.C., Dee R., Hordijk P.L. (2012). Rho GTPase Expression in Human Myeloid Cells. PLoS ONE.

[58] Tolle M., Pawlak A., Schuchardt M., Kawamura A., Tietge U.J., Lorkowski S., Keul P., Assmann G., Chun J., Levkau B. (2008). HDL-Associated Lysosphingolipids Inhibit NAD(P)H Oxidase-Dependent Monocyte Chemoattractant Protein-1 Production. Arterioscler. Thromb. Vasc. Biol..

[59] Holzer M., Wolf P., Curcic S., Birner-Gruenberger R., Weger W., Inzinger M., El-Gamal D., Wadsack C., Heinemann A., Marsche G. (2012). Psoriasis alters HDL composition and cholesterol efflux capacity. J. Lipid Res..

[60] Mao J.Y., Sun J.T., Yang K., Shen W.F., Lu L., Zhang R.Y., Tong X., Liu Y. (2017). Serum amyloid A enrichment impairs the anti-inflammatory ability of HDL from diabetic nephropathy patients. J. Diabetes Complicat..

[61] Cohen G., Rudnicki M., Walter F., Niwa T., Horl W.H. (2001). Glucose-modified proteins modulate essential functions and apoptosis of polymorphonuclear leukocytes. J. Am. Soc. Nephrol..

[62] Cohen G., Raupachova J., Horl W.H. (2013). The uraemic toxin phenylacetic acid contributes to inflammation by priming polymorphonuclear leucocytes. Nephrol. Dial. Transplant..

