# Supplementary material for: Effect of High-Density Lipoprotein from Healthy Subjects and Chronic Kidney Disease Patients on the CD14 Expression on Polymorphonuclear Leukocytes
Source: Int J Mol Sci. 2021 Mar 11;22(6):2830. doi: 10.3390/ijms22062830 (PMC7998954; doi:10.3390/ijms22062830)
Supplement: Supplementary file 1 [file ijms-22-02830-s001.pdf]

International Journal of
Molecular Sciences

MDPI

Table S1. Baseline clinical parameters of study participants (taken from reference [12]).

**Parameter**	**HS**	**CKD3&4**	**HD**
Participants (n)	9	16	15
Gender (m/f)	4/5	12/4	10/5
Age (y)	45.3 (2.8)	64.2 (3.8)**	55.5 (4.5)
Total cholesterol (mg/dl)	187.5 (12.6)	186.1 (7.8)	155.3 (8.5)*,$
Triglycerides (mg/dl)	100.0 (10.8)	161.4 (16.9)*	166.5 (19.7)*
HDL cholesterol (mg/dl)	67.3 (5.7)	49.2 (3.4)*	39.9 (3.0)**
Albumin (g/l)	45.5 (0.8)	40.7 (1.1)**	40.0 (0.9)**
LDL cholesterol (mg/dl)	113.2 (11.5)	104.7 (7.0)	82.3 (7.3)*,$
Creatinine (mg/dl)	0.85 (0.07)	2.3 (0.2)**	10.03 (0.64)**,$$
CRP (mg/dl)	0.08 (0.02)	0.48 (0.13)*	1.19 (0.54)*

Data are shown as mean values (SEM). CKD, chronic kidney disease; CRP, C-reactive protein; HD, hemodialysis; HDL, high-density lipoprotein; HS, healthy subjects; LDL, low-density lipoprotein.

* $p<0.05$, ** $p<0.01$ versus HS; $ $p<0.05$, $$ $p<0.01$ versus CKD3&4

Table S2. Characteristics of study participants (taken from reference [12]).

**Parameter**	**HS**	**CKD3&4**	**HD**
Participants (n)	9	16	15
Diabetes mellitus Type 2 (n)	-	4	4
Hyperlipidemia (n)	-	3	4
Hyperparathyroidism (n)	-	2	2
Osteoporosis (n)	-	2	2
Current medication			
ACE inhibitors (n)	-	3	3
Angiotensin receptor blockers (n)	1	10	5
-blockers (n)	1	10	9
Calcium antagonists (n)	-	9	7
Statins (n)	-	7	2
Glucocorticoids (n)	-	2	2

CKD, chronic kidney disease; HD, hemodialysis; HS, healthy subjects
